# Characterization and Comparison of Milk Fat Globule Membrane Proteins, Whey Protein Concentrate, and Micellar Casein Concentrate

**DOI:** 10.1002/fsn3.71468

**Published:** 2026-01-23

**Authors:** Jiaming Wang, Xiaoguo Ji, Zhongbo Bian, Yuwei Liu, Wenliang Chen, Chuang Fan, Juan Li

**Affiliations:** ^1^ Department of Nutrition, Shanghai Changzheng Hospital Naval Medical University Shanghai China; ^2^ State Key Laboratory of Bioreactor Engineering East China University of Science and Technology Shanghai China; ^3^ School of Public Health/Key Laboratory of Public Health Safety of Ministry of Education Fudan University Shanghai China; ^4^ State Key Laboratory of Dairy Biotechnology, Dairy Research Institute Bright Dairy and Food Co., Ltd. Shanghai China

**Keywords:** label‐free quantification, milk fat globule membrane proteins, proteomics, whey protein

## Abstract

Milk fat globule membrane (MFGM), primarily composed of polar lipids and numerous glycoprotein‐dominated proteins, is an emerging dairy ingredient with considerable application potential. This study systematically characterized the protein composition, structural features, nutritional properties, and digestive characteristics of three bovine milk protein materials: MFGM‐enriched whey protein (MFGMP), whey protein concentrate (WPC), and micellar casein concentrate (MCC). Label‐free quantitative proteomics identified 1025 proteins in MFGMP and 773 in WPC, with 284 differentially expressed proteins (DEPs) between them, including 247 upregulated and 37 downregulated proteins (MFGMP/WPC). Gene Ontology (GO) analysis indicated that the differentially expressed proteins (DEPs) were mainly involved in protein transport, defense response to Gram‐positive bacteria, and negative regulation of endopeptidase activity. Kyoto Encyclopedia of Genes and Genomes (KEGG) pathway analysis revealed enrichment in 45 metabolic pathways, notably complement and coagulation cascades, endocytosis, and regulation of the actin cytoskeleton. Structurally, MFGMP exhibited enhanced stability, characterized by higher α‐helix and lower random coil content compared to WPC and MCC. Nutritionally, while valine was identified as the first limiting amino acid, MFGMP demonstrated a superior amino acid score and a higher essential amino acid index compared to both WPC and MCC, closely aligning with the FAO/WHO reference pattern and establishing it as a high‐quality protein source. During in vitro digestion, MFGMP showed more rapid intestinal degradation with a higher degree of hydrolysis than WPC, demonstrating superior proteolytic accessibility and digestive efficiency. These findings provide mechanistic insights into the distinctive value of MFGMP and establish a scientific basis for developing MFGM‐based functional foods.

## Graphical abstract

This study compared milk fat globule membrane protein (MFGMP) with whey protein concentrate (WPC) and micellar casein concentrate (MCC). The results revealed MFGMP's superior structural stability and amino acid profile, identified its unique proteomic landscape through label‐free quantification, and demonstrated its enhanced in vitro digestibility. These collectively position MFGMP as a high‐quality, functional protein source.

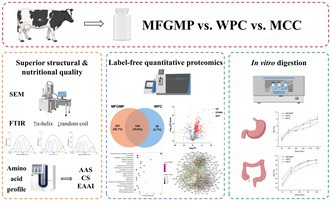

## 1 Introduction

The milk fat globule membrane (MFGM) is a complex trilaminar phospholipoprotein layer surrounding milk fat globules and mainly consists of polar lipids and MFGM proteins (Yao et al. 2024). MFGM proteins account for approximately 25%–70% of the membrane mass and about 1% of total milk proteins, with more than 600 distinct proteins identified to date (Sun et al. 2024). The principal MFGM proteins include mucin 1 (MUC1), mucin 15 (MUC15/PAS III), cluster of differentiation 36 (CD36/PAS IV), butyrophilin (BTN), lactadherin (PAS6/7), xanthine dehydrogenase/oxidase (XDH/XO), adipophilin (ADPH), and fatty acid‐binding protein (FABP) (Manoni et al. 2020). Beyond these major components, MFGM contains numerous low‐abundance proteins that contribute to membrane integrity and physiological regulation. The label‐free proteomic approach has been extensively applied to characterize the MFGM protein profile (Wang et al. 2021).

As a bioactive food ingredient derived from mammary epithelial cell secretions, MFGM‐enriched milk powder is garnering growing interest in the food industry. Multiple clinical trials have explored the safety and efficacy of MFGM‐enriched protein (Hari et al. 2015; Nie et al. 2024; Xie et al. 2025), with findings suggesting potential benefits in promoting brain development and cognitive function (Brink and Lonnerdal 2020), modulating glucose and lipid metabolism and oxidative stress (Demmer et al. 2016; Ji et al. 2024; Rosqvist et al. 2015), as well as enhancing gut health and immune function (Cavaletto et al. 2022; Wu et al. 2022). Despite its promising application as a high‐quality dairy ingredient, the protein composition, structural characteristics, nutritional quality, and digestive behavior of MFGM‐enriched protein remain incompletely elucidated. To address this gap, the present study systematically investigated MFGM‐enriched whey protein (MFGMP), with bovine‐derived whey protein concentrate (WPC) and micellar casein concentrate (MCC) serving as controls. We comprehensively characterized their microstructure, protein secondary structure, and nutritional properties. Label‐free quantitative proteomics was employed to identify compositional differences between MFGMP and WPC and to evaluate their potential functional attributes. Digestive characteristics were assessed using an in vitro simulation model. Our results reveal distinct protein profiles and functional features among these bovine milk protein components, providing a mechanistic foundation for developing MFGM‐derived functional foods.

## 2 Materials and Methods

### 2.1 Materials

Bovine milk protein samples were obtained from commercial sources. MFGM‐enriched whey protein powder (Lacprodan MFGM‐10, Arla Foods Ingredients, Viby J, Denmark; Production dates: 20230108, 20230217, 20230413) was stored cool and dry with an 18‐month shelf life. Whey protein concentrate (WPC 80, Lactalis Ingredients, Savoie, France; Production dates: 20230111, 20230226, 20230325) was stored cool, dry, and protected from sunlight, with an 18‐month shelf life. Micellar casein concentrate (MCC 1900, Vitalus Nutrition Inc., Abbotsford, Canada; Production dates: 20230125, 20230312, 20230426) was stored at 5°C–25°C with ≤ 70% relative humidity, retaining a 24‐month shelf life. Chemical reagents, including dithiothreitol (DTT), acetonitrile, methanol, formic acid, and ammonium formate, were of mass spectrometry (MS) grade. The bicinchoninic acid protein assay kit was provided by Beyotime Biotechnology (Shanghai, China).

### 2.2 Scanning Electron Microscopy (SEM)

The microstructure of protein powders was examined using an SEM (Hitachi S‐3400N, Japan). Samples were mounted on metal stubs with conductive adhesive tape, and loose particles were removed with compressed air. Each sample was sputter‐coated with gold and observed at an accelerating voltage of 5 kV (Romo et al. 2024).

### 2.3 Fourier Transform Infrared Spectroscopy (FTIR)

Protein secondary structures were analyzed using an FTIR spectrometer (Bruker INVENIO S, Germany) based on the amide I absorption band (1600–1700 cm^−1^). Samples were mixed with potassium bromide (KBr) at a 1:10 (w/w) ratio, dried under an infrared lamp, and compressed into pellets at 16 MPa for 4 min. Spectra were collected over the range of 4000–400 cm^−1^ with a resolution of 4 cm^−1^ and 64 scans per spectrum (Wei et al. 2018). Each sample was measured in triplicate. Spectral data were deconvoluted and baseline‐corrected using OMNIC 8.2 (Thermo Fisher Scientific) and PeakFit 4.12 (Systat Software). Secondary structure components were quantified by Gaussian curve fitting of deconvoluted amide I sub‐bands (He et al. 2015).

### 2.4 Amino Acid Composition and Nutritional Assessment

The amino acid profiles of MFGMP, WPC, and MCC were determined using an automated amino acid analyzer (Hitachi LA8080, Japan). The nutritional quality of proteins was evaluated by comparing essential amino acid (EAA) contents per gram of protein with established reference patterns. The amino acid score (AAS) was employed to assess the similarity between the test protein and the FAO/WHO reference pattern (Yang et al. 2018). According to this method, any EAA with an AAS below 100 was identified as a limiting amino acid, and the one with the lowest score was defined as the first limiting amino acid. The AAS was calculated as follows:

(1) $$\begin{array}{cc} \mathrm{AAS}\;=\; & (\mathrm{mg}\;\text{of}\;\mathrm{EAA}\;\text{in}\;1\;g\;\text{test protein}\\ & /\mathrm{mg}\;\text{of the same}\;\mathrm{EAA}\;\text{in}\;1\;g\;\mathrm{FAO}\\ & /\mathrm{WHO}\;\text{reference pattern})\times 100 \end{array}$$

The chemical score (CS) was applied to compare the EAA profiles of test proteins to that of whole egg protein, which is recognized for its high biological value (Shi et al. 2023). EAAs with CS < 1.0 were classified as limiting, with the lowest CS representing the first limiting amino acid. The CS was calculated using the following formula:

(2) $$\begin{array}{cc} \mathrm{CS}\;=\; & (\mathrm{mg}\;\text{of}\;\mathrm{EAA}\;\text{in}\;1\;g\;\text{test protein}\\ & /\mathrm{mg}\;\text{of the same}\;\mathrm{EAA}\;\text{in}\;1\;g\;\text{whole}\;\mathrm{egg}\;\text{protein}) \end{array}$$

The essential amino acid index (EAAI) was calculated as the geometric mean of the ratio of each EAA in the test protein to that in whole egg protein. An EAAI between 86 and 95 indicated a good protein source, 75 to 86 an acceptable source, and below 75 a poor source (Li et al. 2022). A higher EAAI reflected a more balanced amino acid profile and superior protein quality. The EAAI was calculated using the equation:
(3)

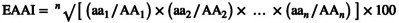

where aa represents the content of an EAA in test protein (mg/g protein), AA represents the content of the same EAA in whole egg protein (mg/g protein), and *n* is the number of EAAs analyzed.

### 2.5 Protein Extraction and Tryptic Digestion

Triplicate samples of MFGMP and WPC were dissolved and centrifuged at 3000 *g* for 15 min at 4°C to remove the fat layer. Proteins were lysed in SDT buffer containing 4% SDS, 100 mM DTT, and 100 mM Tris–HCl (pH 7.6) using ultrasonication. The lysates were centrifuged at 14,000 *g* for 20 min, and the resulting supernatants were filtered through 0.22 μm membranes. Filter‐aided tryptic digestion was conducted at 37°C for 16 h and terminated by the addition of 1% formic acid. Peptides were desalted with C18 solid‐phase extraction columns, lyophilized, and reconstituted in 40 μL of 0.1% formic acid. Peptide concentrations were determined by measuring the absorbance at 280 nm.

### 2.6 LC–MS/MS Analysis

Equal concentrations of peptide solutions were subjected to liquid chromatography using an EASY‐nLC 1200 system (Thermo Fisher Scientific) coupled to an Orbitrap Exploris 480 mass spectrometer (Thermo Fisher Scientific). The mobile phases consisted of solvent A (0.1% formic acid in water) and solvent B (0.1% formic acid in 84% acetonitrile). After equilibration with 95% solvent A, samples were loaded onto a PepMap100 trap column (100 μm × 2 cm, nanoViper C18; Thermo Fisher Scientific) and separated on an EASY‐Column analytical column (75 μm × 10 cm, 3 μm C18‐A2; Thermo Fisher Scientific) at a flow rate of 300 nL/min. The gradient program was 5%–55% solvent B over 0–110 min, 55%–100% solvent B from 110 to 115 min, and 100% solvent B from 115 to 120 min. Mass spectrometry was performed in full‐scan mode with an *m*/*z* range of 350–1600, a capillary temperature of 320°C, and a spray voltage of 2.0 kV.

### 2.7 Protein Identification and Quantification

Raw mass spectrometry data were processed using MaxQuant (v1.6.17) for protein identification and label‐free quantification. Database searches were conducted against the UniProt *Bos taurus* reference proteome (Taxon ID: 9913; 69,735 entries), specifying trypsin as the proteolytic enzyme with a maximum of two missed cleavages. Mass tolerances were set to 20 ppm for precursor ions (MS1) and 0.1 Da for fragment ions (MS/MS). Carbamidomethylation of cysteine was defined as a fixed modification, and methionine oxidation was defined as a variable modification. The false discovery rate (FDR) was controlled using a target‐decoy strategy with reversed sequence databases. Peptide and protein identifications were filtered at an FDR threshold of ≤ 1% in Proteome Discoverer 2.2. Peptide‐spectrum matches (PSMs) with ≥ 99% confidence were accepted, and proteins supported by at least one unique peptide were considered confidently identified.

### 2.8 Screening of Differentially Expressed Proteins (DEPs)

DEPs between the two groups were identified using a combination of Student's *t*‐test (*p* < 0.01) and fold‐change (FC) analysis. FC was defined as the ratio of the mean protein abundance in MFGMP to that in WPC. Proteins with FC > 2 and *p* < 0.01 were considered significantly upregulated, whereas proteins with FC < 0.5 and *p* < 0.01 were classified as significantly downregulated.

### 2.9 Bioinformatics Analysis

DEPs identified between MFGMP and WPC were functionally analyzed using GO annotation (https://geneontology.org/), KEGG pathway enrichment analysis (https://www.genome.jp/kegg/), and the Database for Annotation, Visualization, and Integrated Discovery (DAVID, https://david.ncifcrf.gov/tools.jsp). Protein–protein interaction (PPI) networks were constructed using the STRING database (https://string‐db.org) with a minimum interaction confidence score of 0.400. The resulting networks were visualized in Cytoscape (v3.9.1), and hub proteins were identified using the CytoHubba plugin.

### 2.10 In Vitro Digestion

Electrolyte solutions for simulated digestive fluids were prepared following a previously described method (Chen et al. 2019). Simulated gastric fluid was prepared by dissolving 0.5 g of pepsin (Sigma‐Aldrich, P7000, USA) in 100 mL of simulated gastric electrolyte solution. Simulated intestinal fluid was prepared by dissolving 0.9 g of pancreatin (Sigma‐Aldrich, P7545, USA) in 100 mL of simulated intestinal electrolyte solution, and simulated bile was obtained by dissolving 6.0 g of bile salts (Sigma‐Aldrich, USA) in 100 mL of simulated bile electrolyte solution. All digestive fluids were freshly prepared before use.

The static in vitro digestion procedure followed the INFOGEST protocol (Brodkorb et al. 2019) with minor modifications. Milk protein samples were dissolved in 30 mL of distilled water (10 μg/μL) and preheated at 37°C for 10 min. Simulated gastric fluid containing pepsin was added at a 1:1 (v/v) ratio, and the mixture was incubated at 37°C with shaking at 100 rpm. Aliquots were collected at 0, 30, 60, 90, 120, and 180 min during gastric digestion and immediately adjusted to pH 7.0 with 2 mol/L NaOH to inactivate pepsin. For the intestinal digestion phase, simulated intestinal fluid and bile were mixed at a 2:1 (v/v) ratio. Twenty milliliters of this mixture were added to 20 mL of gastric digesta obtained after 2 h of gastric digestion. The pH was adjusted to 7.0 using 2 mol/L NaOH, followed by incubation at 37°C with shaking at 100 rpm. Aliquots were collected at 15, 30, 60, 90, and 120 min after the initiation of intestinal digestion. The digestion reaction was terminated by heating samples in a boiling water bath at 100°C for 5 min.

### 2.11 Sodium Dodecyl Sulfate‐Polyacrylamide Gel Electrophoresis (SDS‐PAGE)

SDS‐PAGE was conducted using 12% separating gels and 4% stacking gels prepared with a commercial kit following the method of Huang et al. (2022). Gastric digesta were diluted fourfold with deionized water, and intestinal digesta were diluted twofold. Each sample was mixed with 4× loading buffer at a 3:1 (v/v) ratio, boiled for 10 min, and cooled to room temperature. A total of 10 μL of each sample was loaded into individual wells. Electrophoresis was performed at 60 V for stacking and 120 V for separation. Following electrophoresis, gels were stained with Coomassie Brilliant Blue for 2 h and subsequently destained with gentle agitation until the background became clear and transparent for image acquisition.

### 2.12 Degree of Hydrolysis (DH)

DH, defined as the percentage of cleaved peptide bonds in a protein hydrolysate, was determined using the o‐phthalaldehyde (OPA) method (Nielsen et al. 2006). This method quantifies proteolytic degradation by measuring free α‐amino groups released from hydrolyzed peptide bonds (Nehir El et al. 2015). The total number of peptide bonds per gram of protein (*h*_tot_) was calculated based on the amino acid composition. Aliquots collected at various digestion times were diluted, centrifuged at 10,000 *g* for 30 min at 4°C, and the resulting supernatants were filtered through 0.45 μm membranes before analysis.

The OPA reagent was prepared by dissolving 19.05 g of sodium tetraborate decahydrate and 500 mg of sodium dodecyl sulfate (SDS) in 375 mL of deionized water. Separately, 400 mg of OPA was dissolved in 10 mL of absolute ethanol under dark conditions. The two solutions were combined, and 440 mg of DTT was added after complete dissolution. The final mixture was diluted to 500 mL with deionized water in a brown volumetric flask and used immediately.

The standard solution was prepared by dissolving 50 mg of L‐serine in deionized water and diluting to 500 mL. A standard curve was generated using serine standard aliquots of 0, 100, 200, 300, and 400 μL, each adjusted to a final volume of 400 μL with deionized water. Samples were mixed with 3 mL of OPA reagent for 5 s, incubated for 2 min, and the absorbance was measured at 340 nm. The DH was determined by reacting 400 μL of each filtered digestion sample with 3 mL of OPA reagent under identical conditions. Measurements were performed in triplicate, and DH values were calculated using the regression equation derived from the standard curve.

(4) $$\text{Serine}‐{\mathrm{NH}}_{2}=\frac{{\mathrm{OD}}_{\text{sample}}-{\mathrm{OD}}_{\text{blank}}}{{\mathrm{OD}}_{\text{standard}}-{\mathrm{OD}}_{\text{blank}}}\times \frac{0.916\mathrm{meq}.}{L}\times 0.1\times \frac{100}{X}\times P$$

(5) $$h=\left(\text{serine}‐{\mathrm{NH}}_{2}-\beta \right)/\alpha \;\mathrm{meq}./g\;\text{protein}$$

where serine‐NH2 = meq. serine‐NH2 per g protein; *L* = volume of sample; *X* = g sample; *P* = protein% in sample; *α* and *β* are 0.40 and 1.00 respectively

(6) $$\mathrm{DH}\;\left(\%\right)=\frac{h}{h_{\mathrm{tot}}}\times 100$$

where *h*_tot_ is the total number of peptide bonds.

### 2.13 Statistical Analysis

Data visualization was performed using Origin 2022. All measurements were performed in triplicate, and the results are presented as mean ± standard deviation (SD). Statistical analysis was performed using SPSS 26 (IBM Corp., USA). An independent *t*‐test was used for comparisons between two groups. For multiple‐group comparisons, one‐way analysis of variance (ANOVA) was applied, followed by Duncan's multiple range test. Significant differences (*p* < 0.05) among groups are indicated by different lowercase letters.

## 3 Results and Discussion

### 3.1 Microstructural Analysis of MFGMP

**FIGURE 1:**
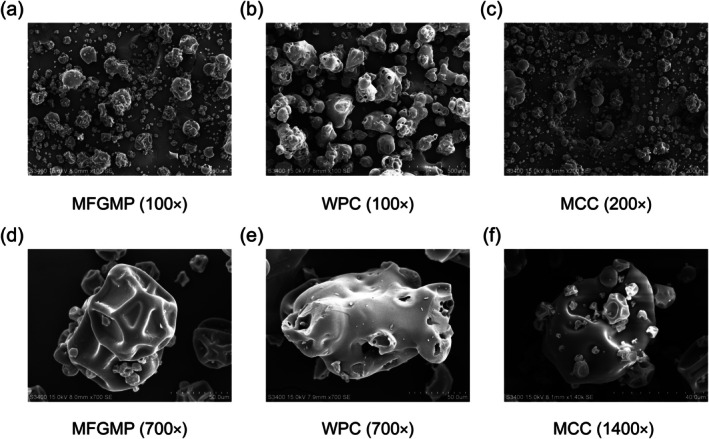
SEM micrographs of milk fat globule membrane protein (MFGMP), whey protein concentrate (WPC), and micellar casein concentrate (MCC). (a) MFGMP (100×); (b) WPC (100×); (c) MCC (200×); (d) MFGMP (700×); (e) WPC (700×); (f) MCC (1400×).

The microstructures of MFGMP, WPC, and MCC were examined using SEM (Figure 1a–f). At 100× magnification, WPC particles appeared the largest, whereas MCC particles were the smallest. At higher magnifications (700× and 1400×), distinct morphological characteristics were observed. MFGMP presented spherical or near‐spherical particles with rough surfaces and prominent wrinkles. WPC exhibited relatively smooth surfaces with small pores, while MCC consisted of fine, irregular particles with coarse surfaces, uneven protrusions, and evident aggregation. The spherical, wrinkled structure of MFGMP is associated with its enhanced emulsification capacity and stability (Li et al. 2024). In contrast, the smooth, porous morphology of WPC correlates with its high solubility and rapid dispersibility. The coarse, aggregated structure of MCC, primarily resulting from the intrinsic hydrophobicity of caseins and the loss of colloidal stability during processing, directly affecting its solubility and hydration properties (Carter et al. 2018). These microstructural differences provide a morphological basis for understanding their distinct behaviors in food systems and during digestion.

### 3.2 Protein Secondary Structure Analysis

The secondary structure of MFGMP, WPC, and MCC was quantified by Gaussian deconvolution of the amide I band (1600–1700 cm^−1^), informed by second‐derivative analysis and presented in the deconvoluted spectra of Figure S1a–c. As summarized in Table 1, the contents of β‐sheet, β‐turn, and β‐antiparallel structures showed no significant differences among the groups (*p* > 0.05). In contrast, the random coil content was significantly lower in MFGMP (14.83 ± 0.68)% than in MCC (17.36 ± 1.20)%, with WPC showing an intermediate value that did not differ significantly from either. Conversely, the α‐helix content was significantly higher in both MFGMP (18.83 ± 0.86)% and WPC (17.69 ± 1.23)% compared to MCC (15.01 ± 1.04)%.

**TABLE 1:** Secondary structural composition of MFGMP, WPC, and MCC (%).

Groups	β—Sheet (1610–1642 cm^−1^)	Random coil (1642–1650 cm^−1^)	α—helix (1650–1660 cm^−1^)	β—turn (1660–1680 cm^−1^)	β—antiparallel (1680–1700 cm^−1^)
MFGMP	34.73 ± 1.59	14.83 ± 0.68^a^	18.83 ± 0.86^a^	22.71 ± 1.04	8.90 ± 0.41
WPC	34.12 ± 2.36	15.64 ± 1.08^ab^	17.69 ± 1.23^a^	23.99 ± 0.83	8.56 ± 0.59
MCC	36.13 ± 1.66	17.36 ± 1.20^b^	15.01 ± 1.04^b^	23.33 ± 0.81	8.17 ± 0.57

*Note:* Different lowercase letters among groups denote significant differences at *p* < 0.05.

Abbreviations: MCC, micellar casein concentrate; MFGMP, milk fat globule membrane protein; WPC, whey protein concentrate.

As established in the literature, ordered structures such as α‐helices and β‐sheets contribute to protein stability, while disordered structures, like β‐turns and random coils, indicate structural flexibility (Cao et al. 2024). A higher degree of structural order supports proper protein folding and defines functional motifs essential for molecular interactions (Yang et al. 2022). Our results revealed that MFGMP and WPC possessed higher α‐helix content than MCC, with MFGMP also exhibiting significantly lower random coil content. These differences directly reflect their inherent protein characteristics. The elevated α‐helix in MFGMP and WPC aligns with the native globular structures of their constituent proteins, stabilized by extensive intramolecular hydrogen bonding. Conversely, the disordered conformation of MCC stems from caseins being intrinsically unstructured proteins, a trait conferred by their high proline content which precludes the stabilization of extensive regular secondary structures (Carver and Holt 2019).

This structural disparity was likely exacerbated by industrial processing (e.g., drying), which disrupts casein micelle stability, promoting aggregation and disorder (Zhong et al. 2025). Consequently, the ordered structure of MFGMP underpins its superior emulsifying functionality, while the prevalent random coils in MCC correlate with its compromised solubility and reduced digestibility (Doiron et al. 2009). Collectively, these findings demonstrate that MFGMP possesses more ordered and stable secondary structures than both WPC and MCC, establishing a direct link between their distinct molecular architectures and their respective functionalities in emulsification, solubility, and digestion.

### 3.3 Amino Acid Composition and Nutritional Quality Evaluation

The amino acid composition underpins protein nutritional quality. According to FAO/WHO guidelines, all three bovine milk proteins demonstrated high essential‐to‐total amino acid ratios (EAA/TAA; 40.74%–44.40%), surpassing the FAO/WHO reference value of 36.00% (Table S1). This confirms that MFGMP, WPC, and MCC are all high‐quality protein sources, consistent with the well‐established nutritional value of bovine milk proteins (Vargas‐Bello‐Perez et al. 2019).

The AAS, CS, and EAAI values are summarized in Table 2. The AAS reflects the alignment between the amino acid composition of a protein and human nutritional needs, with the lowest‐scoring EAA identified as the first limiting amino acid. Valine was the first limiting amino acid in MFGMP, yet its AAS exceeded 100 and was higher than that of the first limiting amino acids in WPC. In contrast, MCC was limited by methionine + cysteine, with a lower AAS (98.97 ± 2.21) than both. These AAS results collectively indicate that MFGMP exhibits the closest conformity to the FAO/WHO reference pattern.

**TABLE 2:** AAS, CS, and EAAI scores of MFGMP, WPC, and MCC.

Amino acid	FAO/WHO reference pattern	Whole egg protein	MFGMP			WPC			MCC		
			Amino acid content (mg/g protein)	AAS	CS	Amino acid content (mg/g protein)	AAS	CS	Amino acid content (mg/g protein)	AAS	CS
Valine	50	66	55.53 ± 0.72	111.07 ± 1.44	0.84 ± 0.01	58.75 ± 0.91	117.50 ± 1.82	0.89 ± 0.01	60.62 ± 0.87	121.24 ± 1.74	0.92 ± 0.01
Isoleucine	40	54	50.33 ± 0.63	125.82 ± 1.57	0.93 ± 0.01	60.88 ± 0.83	152.19 ± 2.08	1.13 ± 0.02	49.48 ± 0.68	123.71 ± 1.70	0.92 ± 0.01
Leucine	70	86	105.86 ± 1.85	151.23 ± 2.64	1.23 ± 0.02	103.02 ± 1.58	147.18 ± 2.26	1.20 ± 0.02	93.61 ± 1.54	133.73 ± 2.20	1.09 ± 0.02
Phenylalanine + Tyrosine	60	93	68.11 ± 1.39	113.52 ± 2.31	0.73 ± 0.01	61.30 ± 0.71	102.17 ± 1.19	0.66 ± 0.01	90.72 ± 1.24	151.20 ± 2.07	0.98 ± 0.01
Methionine + Cystine	35	57	43.39 ± 1.04	123.96 ± 2.98	0.76 ± 0.02	44.27 ± 0.71	126.50 ± 2.02	0.78 ± 0.01	34.64 ± 0.77	98.97 ± 2.21	0.61 ± 0.01
Tryptophan	10	17	12.58 ± 0.39	125.83 ± 3.91	0.74 ± 0.02	12.77 ± 0.39	127.71 ± 3.90	0.75 ± 0.02	10.72 ± 0.43	107.22 ± 4.30	0.63 ± 0.03
Threonine	40	47	62.47 ± 1.46	156.18 ± 3.65	1.33 ± 0.03	65.56 ± 1.74	163.90 ± 4.35	1.39 ± 0.04	41.24 ± 1.09	103.09 ± 2.73	0.88 ± 0.02
Lysine	55	70	88.07 ± 1.31	160.12 ± 2.38	1.26 ± 0.02	86.42 ± 1.25	157.13 ± 2.27	1.23 ± 0.02	77.53 ± 1.11	140.96 ± 2.02	1.11 ± 0.02
EAAI					95.03 ± 0.34			97.18 ± 0.84			87.14 ± 0.84

Abbreviations: AAS, amino acid score; CS, chemical score; EAAI, essential amino acid index; MCC, micellar casein concentrate; MFGMP, milk fat globule membrane protein; WPC, whey protein concentrate.

The CS assesses protein quality by comparing the EAA composition of a protein with that of whole egg protein, recognized as an ideal standard for human absorption. MFGMP exhibited the lowest CS for phenylalanine + tyrosine but showed higher values for lysine, threonine, and leucine, following a pattern similar to WPC. MCC, however, was limiting in most EAAs except for leucine and lysine, with methionine + cysteine being the most constrained (CS = 0.61). The EAAI confirmed the high quality of all proteins (> 86), with WPC and MFGMP being comparable and superior to MCC.

The robust EAAI of MFGMP is particularly noteworthy. It suggests that its diverse protein profile, encompassing both membrane‐specific proteins and soluble whey proteins, contributes to a nutritionally balanced and complementary amino acid matrix (Xie et al. 2025). In summary, these indices affirm that MFGMP is a nutritionally competitive high‐quality protein, with an amino acid profile comparable to or surpassing that of WPC and distinctly superior to MCC.

### 3.4 Protein Compositions of MFGMP and WPC

**FIGURE 2:**
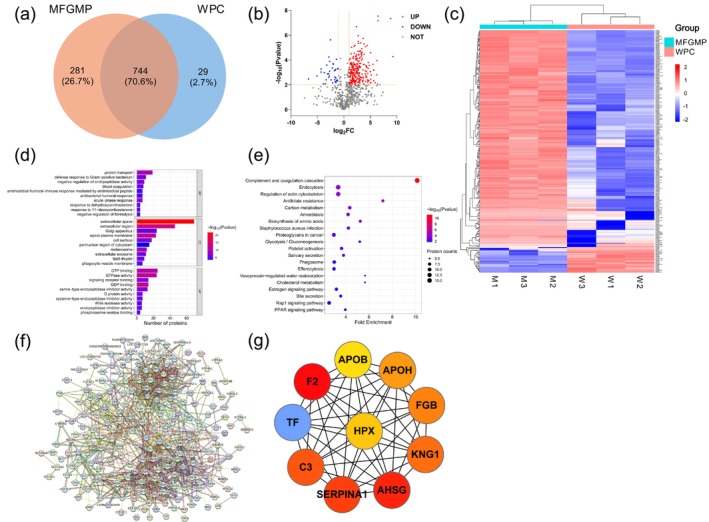
(a) Venn diagram of proteins identified in MFGMP and WPC. (b) Volcano plot of DEPs in MFGMP and WPC. (c) Heatmap of DEPs in MFGMP and WPC. (d) GO enrichment analysis of DEPs in MFGMP and WPC. (e) KEGG pathway enrichment analysis of DEPs in MFGMP and WPC. (f) PPI network analysis of DEPs in MFGMP and WPC. (g) Hub protein networks of the top 10 MCC‐scored DEPs. MFGMP, milk fat globule membrane protein; WPC, whey protein concentrate; DEPs, differentially expressed proteins.

A total of 1025 and 773 proteins were identified in MFGMP and WPC, respectively, with 744 proteins shared between the two groups. MFGMP contained 281 unique proteins, whereas WPC contained only 29 (Figure 2a). The 20 most abundant proteins in MFGMP (Table 3) and WPC (Table S2) are listed. Several high‐abundance proteins were detected in both samples, including β‐lactoglobulin, albumin, glycosylation‐dependent cell adhesion molecule 1, lactotransferrin, butyrophilin subfamily 1 member A1, Ig‐like domain‐containing protein, milk fat globule‐EGF factor 8 protein, enterotoxin‐binding glycoprotein PP20K, xanthine dehydrogenase/oxidase, and α‐lactalbumin. Both α‐S1‐casein and α‐S2‐casein were also present in MFGMP and WPC, likely due to casein adsorption onto MFGMP fragments generated during industrial processing. Procedures such as refrigeration and high‐speed centrifugation may disrupt milk fat globules, releasing membrane fragments that bind residual caseins, thereby preventing complete separation. The observed phenomenon aligns with previous findings (Holzmüller et al. 2016; Sun et al. 2020; Zhao et al. 2023).

**TABLE 3:** The main proteins in MFGMP.

UniProt accession	Protein description	Log_2_ peak area intensity	Coverage (%)	MW (kDa)	PI
A0A4W2DRY6	Beta‐lactoglobulin	34.75	65	26.2	6.98
A0A140T897	Albumin	34.48	73	69.3	6.18
P80195	Glycosylation‐dependent cell adhesion molecule 1	34.20	48	17.1	6.68
B3VTM3	Lactotransferrin	33.87	65	78.0	8.32
B5B0D4	Major allergen beta‐lactoglobulin	33.31	79	20.0	4.94
P18892	Butyrophilin subfamily 1 member A1	33.11	58	59.2	5.20
A0A3Q1M3L6	Ig‐like domain‐containing protein	32.52	51	40.5	5.34
F1MXX6	Milk fat globule EGF and factor V/VIII domain containing	32.49	60	47.8	7.15
Q9TRB9	Enterotoxin‐binding glycoprotein PP20K	32.19	40	2.3	8.73
F1MUT3	Xanthine dehydrogenase/oxidase	32.06	41	146.7	7.68
A0A452DI34	Alpha‐lactalbumin	32.03	34	19.3	5.20
A6QM09	Ig‐like domain‐containing protein	31.68	47	24.7	7.61
A6QNW3	PIGR protein	31.40	44	82.5	7.28
P02662	Alpha‐S1‐casein	31.09	49	24.5	5.02
G3MXZ0	Lactoperoxidase	31.06	56	82.7	8.54
G3X6N3	Serotransferrin	31.00	64	77.7	7.17
G5E513	Ig‐like domain‐containing protein	30.86	59	48.1	5.59
C0LSL0	Heart fatty acid‐binding protein	30.85	47	14.8	7.34
A0A3Q1LZU0	Secreted phosphoprotein 1	30.65	22	37.1	4.72
P26201	Platelet glycoprotein 4	30.44	16	52.9	8.10

Abbreviation: MFGMP, milk fat globule membrane protein.

Milk proteins display considerable compositional complexity, and increasing attention has been directed toward milk fat globule membrane proteins due to their distinctive profiles and biological functions. Prior label‐free proteomic analyses have revealed interspecies variation in MFGM protein composition. Chen et al. (2023) identified 1292 MFGM and 686 whey proteins across four bovid species. Han et al. (2022) reported 1133 MFGM and 627 whey proteins in camel milk, and Lv et al. (2024) characterized 773 MFGM and 596 whey proteins in equine milk. The MFGM proteome identified in the present study extends current knowledge of bovine milk protein composition and provides a basis for developing MFGM‐enriched formulations for food applications.

### 3.5 Differentially Expressed Proteins in MFGMP and WPC

Although MFGMP and WPC shared a substantial number of proteins, their abundance profiles differed markedly, reflecting potential variations in nutritional and functional properties. To identify DEPs between the two groups, thresholds were set at *p* < 0.01 and FC > 2.0 or < 0.5, resulting in 284 DEPs. As shown in the volcano plot (Figure 2b), 247 proteins were upregulated (red) and 37 were downregulated (blue) in MFGMP compared with WPC. The heatmap (Figure 2c) illustrates the hierarchical clustering of these significant DEPs, demonstrating clear separation between the two protein fractions.

Compared with WPC, MFGMP contained a greater number of significantly upregulated proteins (Table 4). Prominent upregulated proteins included IST1 homolog, angiogenin‐1, carboxypeptidase D, alpha‐1‐antiproteinase, Ig‐like domain‐containing protein, and annexin A2. In contrast, downregulated proteins were mainly palmitoyl‐protein thioesterase, CutA divalent cation tolerance homolog, tripeptidyl‐peptidase 1, beta‐1,4‐galactosyltransferase 1, carboxypeptidase B2, and cystatin E/M.

**TABLE 4:** The significant DEPs in MFGMP and WPC.

UniProt accession	Protein description	Molar mass/kDa	*p*	Fold change (MFGMP/WPC)
F1MXJ5	IST1 homolog	39.8	< 0.01	622.05
P10152	Angiogenin‐1	17.0	< 0.01	458.37
E1BLR9	Carboxypeptidase D	152.5	< 0.01	88.16
P34955	Alpha‐1‐antiproteinase	46.1	< 0.01	87.27
G3MZE0	Ig‐like domain‐containing protein	14.4	< 0.01	82.30
P04272	Annexin A2	38.6	< 0.01	75.57
A0A3Q1M2Y5	Protein tweety homolog	79.4	< 0.01	73.99
E1BLF1	PATJ crumbs cell polarity complex component	207.0	< 0.01	60.65
E1BDE3	Protein‐serine/threonine phosphatase	29.1	< 0.01	51.04
P63097	Guanine nucleotide‐binding protein G(i) subunit alpha‐1	40.3	< 0.01	49.66
A0A3Q1M5Z8	Palmitoyl‐protein hydrolase	35.6	< 0.01	0.01
F1N5T0	CutA divalent cation tolerance homolog	18.9	< 0.01	0.03
A0A3Q1N178	Tripeptidyl‐peptidase 1	62.3	< 0.01	0.05
P08037	Beta‐1,4‐galactosyltransferase 1	44.8	< 0.01	0.05
Q2KIG3	Carboxypeptidase B2	48.8	< 0.01	0.07
Q5DPW9	Cystatin E/M	16.3	< 0.01	0.08
A5LIP3	Carboxypeptidase	53.9	< 0.01	0.09
Q1RMI2	Ras homolog family member G	21.3	< 0.01	0.10
B0JYL8	Cofilin‐1	18.5	< 0.01	0.10
A0A3Q1N1B0	Serine peptidase inhibitor, Kunitz type 3	16.0	< 0.01	0.12

Abbreviations: DEPs, differentially expressed proteins; MFGMP, milk fat globule membrane protein; WPC, whey protein concentrate.

### 3.6 Functional Attributes of Differentially Expressed Proteins in MFGMP and WPC

GO functional annotation was conducted for the 284 DEPs shared between MFGMP and WPC across three main categories: biological process (BP), cellular component (CC), and molecular function (MF) (Figure 2d). In the BP category, DEPs were predominantly enriched in protein transport, defense response to Gram‐positive bacteria, negative regulation of endopeptidase activity, blood coagulation, antimicrobial humoral immune response mediated by antimicrobial peptides, antibacterial humoral response, acute‐phase response, responses to dehydroepiandrosterone and 11‐deoxycorticosterone, and negative regulation of fibrinolysis. For the CC category, enrichment was mainly observed in the extracellular space, extracellular region, Golgi apparatus, apical plasma membrane, cell surface, perinuclear cytoplasmic region, melanosome, extracellular exosome, lipid droplet, and phagocytic vesicle membrane. In the MF category, significant enrichment was detected for GTP binding, GTPase activity, GDP binding, signaling receptor binding, serine‐type and cysteine‐type endopeptidase inhibitor activity, G protein activity, endopeptidase inhibitor activity, RNA nuclease activity, and phosphoserine residue binding.

### 3.7 KEGG Enrichment Identifies Key Pathways for Differentially Expressed Proteins

KEGG pathway enrichment analysis was conducted to elucidate the biological pathways and potential functions associated with the 284 DEPs between MFGMP and WPC. These proteins were mapped to 45 metabolic pathways, with the top 20 presented in Figure 2e. Major enriched pathways included complement and coagulation cascades, endocytosis, regulation of the actin cytoskeleton, antifolate resistance, carbon metabolism, amoebiasis, biosynthesis of amino acids, *Staphylococcus aureus* infection, proteoglycans in cancer, and glycolysis/gluconeogenesis.

Complement and coagulation cascades are recognized as central immune‐related pathways. The innate immune response plays a fundamental role in defense against viral infections through the coordinated action of the complement system, coagulation cascade, and natural antibodies. These systems function within a precisely regulated network that maintains immune equilibrium and prevents tissue injury caused by excessive activation (Maloney et al. 2020). The complement system acts as a primary effector of innate immunity by promoting microbial lysis, apoptotic cell clearance, and inflammatory responses (Bajic et al. 2015). Activation of the coagulation cascade further enhances immune protection through fibrin formation and platelet activation, reinforcing antiviral defense (Antoniak and Mackman 2014). Endocytosis, a process responsible for the internalization of macromolecules, contributes to both immunomodulatory and nutrient transport functions of milk proteins. Immunoglobulins and lactoferrin, for example, are absorbed by intestinal and immune cells through receptor‐mediated endocytosis, facilitating intracellular signaling and nutrient transfer (Jiang et al. 2011). In mammary epithelial cells, this mechanism supports milk secretion by promoting membrane recycling and exosome biogenesis, reflecting its involvement in transmembrane transport and intercellular communication (Kusuma et al. 2016). Regulation of the actin cytoskeleton, essential for T cell, B cell, and macrophage function, modulates immune responses through cytoskeletal remodeling (Burbage and Keppler 2018). The enrichment of DEPs in pathways related to metabolism, human diseases, and organismal systems suggests that MFGMP proteins play a crucial role in conferring passive immunity to neonatal mammals, providing protection against pathogenic infections (Raza et al. 2021; Timby et al. 2015).

### 3.8 PPI Network Identifies Key Regulatory Hubs

Proteins act as primary molecular effectors of biological processes and participate in nearly all cellular functions, including signal transduction, gene expression regulation, metabolic activity, cytoskeletal organization, and cell division and differentiation. Such complex processes depend on the coordinated actions of multiple proteins rather than isolated molecules. PPI network analysis of the 284 DEPs is essential for elucidating the biological roles of milk fat globule membrane proteins and identifying potential regulatory hubs. A total of 239 DEPs with interaction confidence scores ≥ 0.400 were incorporated into a PPI network comprising 968 interactions (Figure 2f). Annexin A2 (ANXA2) exhibited the highest connectivity, interacting with 35 proteins, followed by apolipoprotein B (APOB) and endoplasmic reticulum chaperone BiP (HSPA5), each interacting with 34. Complement C3 (C3) and phosphopyruvate hydratase (ENO1) each engaged in 30 interactions, while alpha‐2‐HS‐glycoprotein (AHSG) and calreticulin (CALR) showed 28. Beta‐2‐glycoprotein 1 (APOH) and alpha‐1‐antiproteinase (SERPINA1) exhibited 26 interactions, and prothrombin (F2) was linked to 25. The PPI network was significantly enriched in pathways such as complement and coagulation cascades, galactose metabolism, SNARE‐mediated vesicular transport, cholesterol metabolism, and ferroptosis.

Hub proteins were ranked using the Maximal Clique Centrality (MCC) algorithm, with the top 10 displayed in Figure 2g (nodes in red indicate higher MCC scores). Prothrombin (F2) achieved the highest MCC score, followed by alpha‐2‐HS‐glycoprotein (AHSG), alpha‐1‐antiproteinase (SERPINA1), complement C3 (C3), kininogen‐1 (KNG1), fibrinogen beta chain (FGB), beta‐2‐glycoprotein 1 (APOH), serotransferrin (TF), hemopexin (HPX), and apolipoprotein B (APOB). As a central hub in the PPI network, complement C3 is pivotal to the innate immune system, forming a crucial host defense mechanism to detect and clear potential pathogens (Delanghe et al. 2014; Ricklin et al. 2016). While complement proteins in colostrum are reported to supply passive immunity to infants (Castillo‐Lopez et al. 2025; Zhang et al. 2015), the high connectivity and central ranking of C3 within the MFGMP network provide novel evidence for its potential role in this material. This suggests that a complete complement functional axis is embedded within the molecular architecture of MFGMP, offering a plausible mechanism for immune modulation and thereby validating the significant enrichment of the complement and coagulation cascades pathway at the systems level. The constituent proteins of this pathway, including the central component C3, the core zymogen F2, the critical inhibitor SERPINA1, and key regulators AHSG, KNG1, and FGB, collectively exhibit high connectivity and top MCC rankings, converging to form a synergistic functional module. The architecture of this module integrates potent activators (C3, F2) with a major inhibitor (SERPINA1), revealing an intrinsic framework that enables balanced immunomodulatory and proteolytic regulation in MFGMP. Collectively, this provides systems‐level evidence for MFGMP's potential bioactivity in mediating host defense and inflammatory homeostasis.

### 3.9 Comparative Digestive Profiles of MFGMP, WPC, and MCC

**FIGURE 3:**
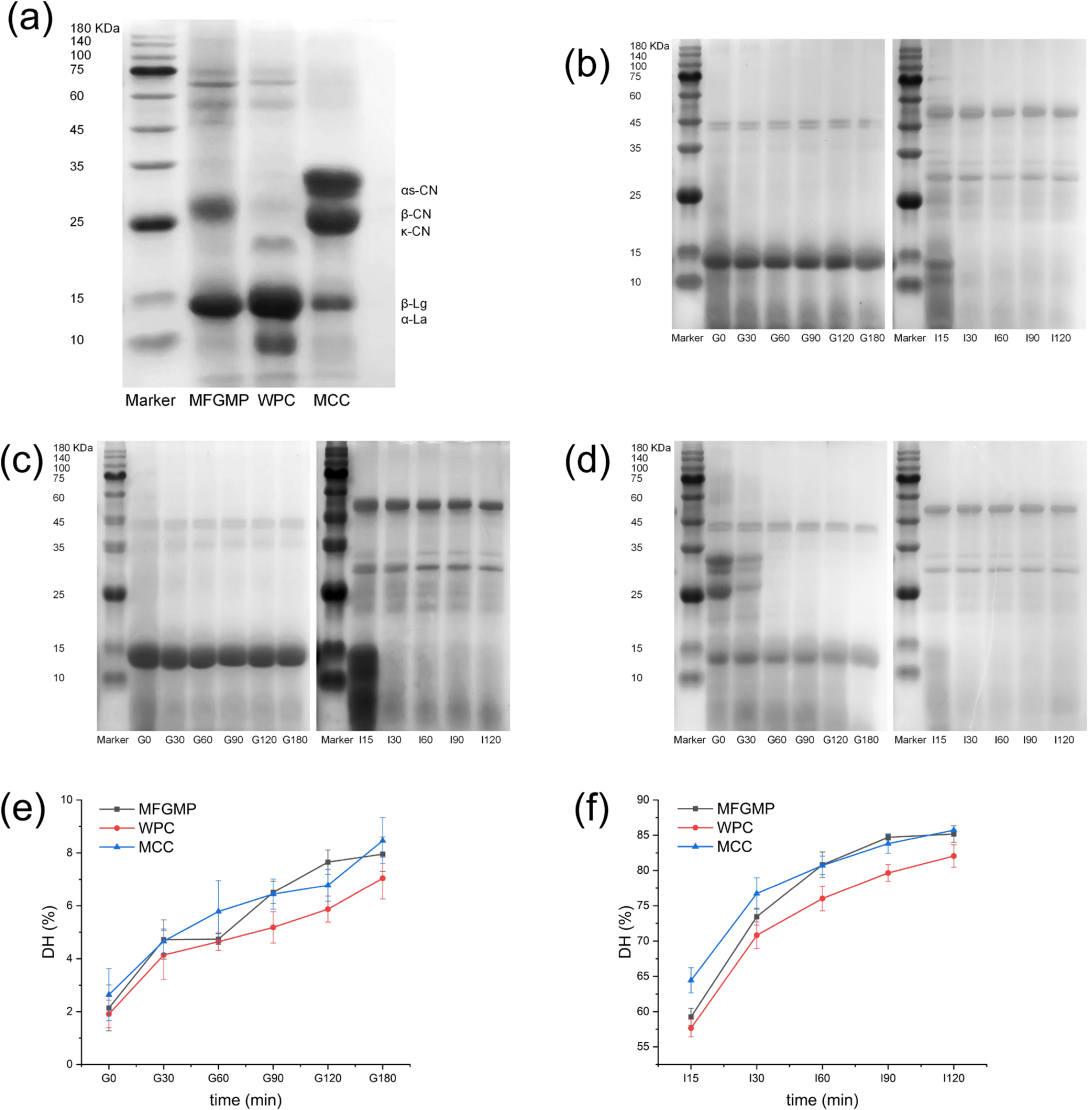
(a) SDS‐PAGE protein profiles of MFGMP, WPC, and MCC. SDS‐PAGE profiles of (b) MFGMP, (c) WPC, and (d) MCC during gastric/intestinal digestion. DH during in vitro gastric digestion (e) and intestinal digestion (f). G0, undigested; G30, 30 min gastric digestion; I15, 15 min intestinal digestion; MCC, micellar casein concentrate; MFGMP, milk fat globule membrane protein; WPC, whey protein concentrate.

SDS‐PAGE revealed distinct electrophoretic profiles among the three bovine milk protein materials under native conditions (Figure 3a) and dynamic compositional changes during in vitro digestion (Figure 3b–d). MFGMP displayed prominent bands corresponding to β‐lactoglobulin (β‐Lg, ~18 kDa) and α‐lactalbumin (α‐La, ~14 kDa), accompanied by several faint high‐molecular‐weight bands (> 70 kDa) attributable to lactoferrin and serum albumin. WPC exhibited pronounced bands for β‐Lg and α‐La, along with other whey‐derived proteins. MCC was dominated by intense bands representing αs‐casein (αs‐CN, ~32 kDa), κ‐casein (κ‐CN, ~19 kDa), and β‐casein (β‐CN, ~24 kDa). Residual β‐Lg was also detected in MCC, likely resulting from heat‐induced disulfide bonding between κ‐CN on the casein micelle surface and β‐Lg during processing. Such interactions promote aggregation, and the resulting complexes adhere tightly to micellar surfaces, making the bound β‐Lg difficult to eliminate (Anema and Li 2000).

During gastric digestion, casein bands gradually weakened, indicating ongoing proteolysis, whereas whey protein bands, primarily β‐Lg, remained largely intact, confirming their low gastric digestibility. In the intestinal phase, casein bands diminished markedly within 15 min, reflecting rapid hydrolysis, while whey protein bands (β‐Lg and α‐La) were completely digested within 30 min, demonstrating efficient degradation by trypsin and chymotrypsin.

These observations align with previous reports (Lavoisier et al. 2023; Lorieau et al. 2018), where whey proteins are identified as fast‐digesting, linked to rapid postprandial aminoacidemia (Schmedes et al. 2018), while caseins are slow‐digesting due to their tendency to form curds in the stomach, which delays gastric emptying and prolongs amino acid release (Horstman and Huppertz 2023). The compact globular structure of β‐Lg confers resistance to pepsin hydrolysis (da Silva et al. 2023), while the smaller whey protein curds improve solubility, facilitate intestinal transit, and accelerate amino acid absorption (Traylor et al. 2019). During intestinal digestion, caseins exhibited greater susceptibility to tryptic hydrolysis than whey proteins. This difference arises from structural variations: caseins possess an open tertiary structure enhanced by phosphorylation, increasing their accessibility to proteolytic enzymes (Holt et al. 2013). Conversely, whey proteins are rich in sulfur‐containing amino acids (e.g., cysteine, methionine, lysine, threonine, and tryptophan), which promote disulfide bond formation and yield compact conformations that restrict enzymatic cleavage (Nguyen et al. 2015).

### 3.10 Distinct DH of Bovine Milk Proteins During in Vitro Digestion

The degree of hydrolysis was quantified by measuring the release of free amino groups generated during proteolysis using the OPA assay. Under alkaline conditions, primary amines react with o‐phthalaldehyde to form isoindole derivatives that exhibit maximal absorbance at 340 nm. The total peptide bond hydrolysis constants (*h*_tot_) for the three milk proteins are provided in Table S3.

During the gastric phase (Figure 3e), all samples displayed low initial hydrolysis. As digestion progressed, MFGMP showed significantly higher DH values than WPC at 90, 120, and 180 min (*p* < 0.05). After 180 min of gastric digestion, the DH of MFGMP reached (7.95 ± 0.65)%, showing only a slight increase beyond 120 min, suggesting a plateau in late‐stage gastric proteolysis. MCC maintained higher DH values than WPC between 60 and 180 min, reaching (8.46 ± 0.87)% by the end of the gastric phase. These findings corresponded well with electrophoretic patterns, where the whey protein fraction (mainly β‐lactoglobulin and α‐lactalbumin) showed pronounced resistance to pepsin hydrolysis (Mandalari et al. 2009). Consequently, both MFGMP and WPC exhibited limited gastric digestibility but remained soluble, whereas MCC formed poorly soluble curds that underwent faster proteolysis, promoting their retention in the stomach and delaying subsequent intestinal digestion and amino acid absorption.

In the intestinal phase (Figure 3f), DH increased rapidly within the first 15 min across all samples, confirming the small intestine as the primary site for casein and whey protein hydrolysis. MFGMP and WPC displayed comparable DH values at 15 and 30 min, but MFGMP exhibited significantly higher values between 60 and 120 min (*p* < 0.05). MCC initially showed higher DH than the other groups at 15 min, and by 120 min, both MCC and MFGMP surpassed WPC (*p* < 0.05). The enhanced digestibility of MFGMP may be attributed to residual lipids that facilitate enzyme accessibility, as β‐lactoglobulin adsorbed on fat droplet interfaces exhibits greater susceptibility to proteolytic cleavage than in the soluble phase (Macierzanka et al. 2009). Beyond this, the distinct digestion kinetics of MFGMP, which shares an initial rate with WPC but attains a final hydrolysis extent akin to MCC, are fundamentally linked to its composition as an MFGM‐rich material. The native architecture of MFGM, comprising specific proteins (e.g., Mucin‐1, Butyrophilin) intimately associated with phospholipids, likely modulates proteolysis by presenting interfacial barriers that initially slow enzyme access, while subsequently providing a sustained lipid‐protein matrix for digestion (Y. Zhao et al. 2024). This comparative analysis elucidates how MFGMP confers a hybrid digestive functionality, intermediate between whey and casein profiles. By systematically comparing these three materials, our study provides novel mechanistic insight into the structure–function relationship of MFGMP, highlighting its potential for nutritional applications where tailored amino acid release is desired.

## 4 Conclusions

This study establishes that MFGMP possesses distinctive characteristics that support its potential as a high‐value functional ingredient. Structural analysis revealed that MFGMP possesses enhanced molecular stability, characterized by a higher α‐helix and a lower random coil content compared to WPC and MCC. Nutritionally, MFGMP qualifies as a high‐quality protein based on its superior AAS and higher EAAI compared to both WPC and MCC, with these values closely aligning with the FAO/WHO reference pattern. Proteomic profiling further distinguished MFGMP, identifying 1025 proteins compared to 773 in WPC. Among these, DEPs were significantly enriched in 45 metabolic pathways, including complement and coagulation cascades, endocytosis, and actin cytoskeleton regulation, suggesting their potential role in immune regulation and tissue homeostasis. During in vitro digestion, MFGMP exhibited excellent digestive behavior, characterized by more rapid intestinal degradation and a higher degree of hydrolysis versus WPC, indicating superior proteolytic accessibility and digestive efficiency.

Several limitations of this study should be considered. First, the in vitro digestion model, while controlled, cannot fully replicate the complex physiological environment of the human gastrointestinal tract, including peristalsis and the role of the gut microbiota. Second, as a commercial ingredient, the protein composition of MFGMP may vary with milk source and processing, which could affect the generalizability of the findings. Third, while GO and KEGG analyses provide valuable insights into potential functional pathways, the bioinformatic predictions require further mechanistic validation through targeted molecular and cellular assays to establish direct causative links. Collectively, these integrated findings position MFGMP as a promising ingredient for functional foods targeting immune support and metabolic health. Future work, including animal studies and human clinical trials, is essential to validate these benefits in vivo and explore its therapeutic applications.

## Author Contributions

J.W.: investigation, writing – original draft, project administration. X.J.: conceptualization, supervision, writing – review and editing. Z.B.: formal analysis, visualization, data curation. Y.L.: methodology, investigation. W.C.: resources, funding acquisition. C.F.: validation, investigation. J.L.: conceptualization, methodology, writing – review and editing.

## Funding

This work was supported by grants from the Scientific Research Project of the Shanghai Municipal Science and Technology Commission (24Y12801100), Shanghai Sailing Program (23YF1409800) and the Fudan University‐Industry Joint Research Project (SGF201425).

## Conflicts of Interest

The authors declare no conflicts of interest.

## Supporting information


**Data S1:** fsn371468‐sup‐0001‐Supinfo.docx.

## Data Availability

Data sets generated during the study are available from the corresponding author upon request.
