# Supplementary material for: Characterization and Comparison of Milk Fat Globule Membrane Proteins, Whey Protein Concentrate, and Micellar Casein Concentrate
Source: Food Sci Nutr. 2026 Jan 23;14(1):e71468. doi: 10.1002/fsn3.71468 (PMC12828347; doi:10.1002/fsn3.71468)
Supplement: Supplementary file 1 — Data S1: fsn371468‐sup‐0001‐Supinfo.docx. [file FSN3-14-e71468-s001.docx]

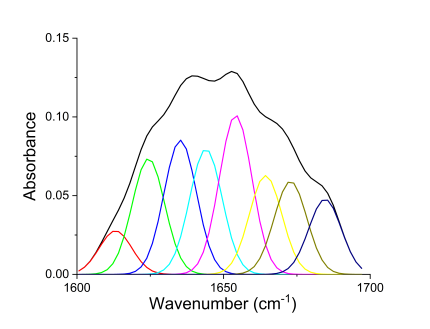

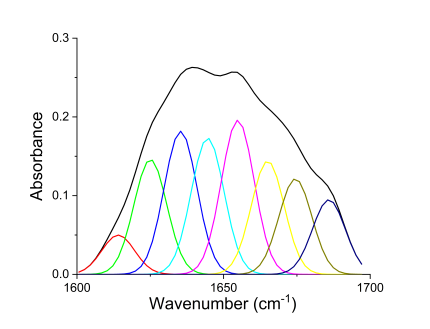

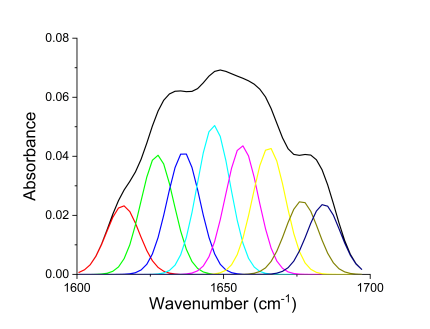


(a) (b) (c)

**Figure S1** FTIR spectra and curve-fitting results of the amide I band for (a) MFGMP, (b) WPC, and (c) MCC. MFGMP, milk fat globule membrane protein; WPC, whey protein concentrate; MCC, micellar casein concentrate.

**Table S1** Amino acid content of MFGMP, WPC, and MCC (Mean ± SD, mg/g protein)

| Amino acid | MFGMP | WPC | MCC |
| --- | --- | --- | --- |
| Valine^*^ | 55.53 ± 0.72 | 58.75 ± 0.91 | 60.62 ± 0.87 |
| Isoleucine^*^ | 50.33 ± 0.63 | 60.88 ± 0.83 | 49.48 ± 0.68 |
| Leucine^*^ | 105.86 ± 1.85 | 103.02 ± 1.58 | 93.61 ± 1.54 |
| Phenylalanine^*^ | 38.18 ± 0.52 | 33.21 ± 0.47 | 45.77 ± 0.39 |
| Tyrosine | 29.93 ± 0.91 | 28.1 ± 0.56 | 44.95 ± 0.97 |
| Methionine^*^ | 22.56 ± 0.58 | 23.41 ± 0.69 | 28.45 ± 0.72 |
| Cysteine | 20.82 ± 0.46 | 20.86 ± 0.48 | 6.19 ± 0.26 |
| Tryptophan^*^ | 12.58 ± 0.39 | 12.77 ± 0.39 | 10.72 ± 0.43 |
| Threonine^*^ | 62.47 ± 1.46 | 65.56 ± 1.74 | 41.24 ± 1.09 |
| Lysine^*^ | 88.07 ± 1.31 | 86.42 ± 1.25 | 77.53 ± 1.11 |
| Aspartic acid | 94.58 ± 1.65 | 96.64 ± 1.63 | 69.69 ± 0.93 |
| Serine | 58.13 ± 1.22 | 50.23 ± 1.05 | 51.55 ± 1.28 |
| Glutamic acid | 170.93 ± 2.47 | 185.61 ± 3.02 | 217.73 ± 3.45 |
| Glycine | 23.43 ± 0.49 | 18.31 ± 0.55 | 17.32 ± 0.34 |
| Alanine | 47.72 ± 0.77 | 48.11 ± 0.57 | 31.34 ± 0.38 |
| Histidine | 23.43 ± 0.47 | 17.88 ± 0.49 | 25.98 ± 0.51 |
| Arginine | 39.48 ± 0.65 | 25.54 ± 0.67 | 32.99 ± 0.53 |
| Proline | 55.97 ± 0.63 | 64.71 ± 1.02 | 94.85 ± 1.52 |
| EAA/TAA（%） | 43.56 ± 0.13 | 44.40 ± 0.47 | 40.74 ± 0.28 |

MFGMP, milk fat globule membrane protein; WPC, whey protein concentrate; MCC, micellar casein concentrate; EAA, essential amino acid; TAA, total amino acid; * EAA.

**Table S2** The main proteins in WPC

| UniProt accession | Protein description | Log_2_ peak area intensity | Coverage (%) | MW  (kDa) | pl |
| --- | --- | --- | --- | --- | --- |
| A0A4W2DRY6 | Beta-lactoglobulin | 35.58 | 70 | 26.2 | 6.98 |
| A0A140T897 | Albumin | 34.41 | 69 | 69.3 | 6.18 |
| A0A452DI34 | Alpha-lactalbumin | 34.07 | 34 | 19.3 | 5.20 |
| P80195 | Glycosylation-dependent cell adhesion molecule 1 | 33.80 | 51 | 17.1 | 6.68 |
| B5B0D4 | Major allergen beta-lactoglobulin | 33.41 | 76 | 20.0 | 4.94 |
| Q9TRB9 | Enterotoxin-binding glycoprotein PP20K | 33.37 | 40 | 2.3 | 8.73 |
| B3VTM3 | Lactotransferrin | 33.15 | 61 | 78.0 | 8.32 |
| A0A3Q1M3L6 | Ig-like domain-containing protein | 32.95 | 42 | 40.5 | 5.34 |
| P02662 | Alpha-S1-casein | 32.61 | 45 | 24.5 | 5.02 |
| P02663 | Alpha-S2-casein | 32.22 | 38 | 26.0 | 8.43 |
| P18892 | Butyrophilin subfamily 1 member A1 | 31.67 | 49 | 59.2 | 5.20 |
| A6QNW3 | PIGR protein | 31.29 | 42 | 82.5 | 7.28 |
| Q2UVX4 | Complement C3 | 31.17 | 51 | 187.1 | 6.84 |
| F1MXX6 | Milk fat globule EGF and factor V/VIII domain containing | 30.82 | 54 | 47.8 | 7.15 |
| G5E513 | Ig-like domain-containing protein | 30.81 | 57 | 48.1 | 5.59 |
| F1MUT3 | Xanthine dehydrogenase/oxidase | 30.78 | 35 | 146.7 | 7.68 |
| A0A3Q1N9Y5 | Sulfhydryl oxidase | 30.64 | 25 | 86.1 | 8.91 |
| G3MXZ0 | Lactoperoxidase | 30.45 | 51 | 82.7 | 8.54 |
| A0A3Q1LZU0 | Secreted phosphoprotein 1 | 30.11 | 20 | 37.1 | 4.72 |
| C0LSL0 | Heart fatty acid-binding protein | 29.36 | 42 | 14.8 | 7.34 |

WPC, whey protein concentrate.

**Table S3** *h*_tot_ of MFGMP, WPC, and MCC (Mean ± SD)

| Amino acid | MW  (kDa) | MFGMP | | | WPC | | | MCC | |
| --- | --- | --- | --- | --- | --- | --- | --- | --- | --- |
|  |  | Amino acid content (mg/g protein) | Peptide Bond Millimoles (mmol/g) | Amino acid content (mg/g protein) | | Peptide Bond Millimoles (mmol/g) | Amino acid content (mg/g protein) | | Peptide Bond Millimoles (mmol/g) |
| Valine | 117.20 | 55.53 ± 0.72 | 0.474 ± 0.006 | 58.75 ± 0.91 | | 0.501 ± 0.008 | 60.62 ± 0.87 | | 0.517 ± 0.007 |
| Isoleucine | 131.18 | 50.33 ± 0.63 | 0.384 ± 0.005 | 60.88 ± 0.83 | | 0.464 ± 0.006 | 49.48 ± 0.68 | | 0.377 ± 0.005 |
| Leucine | 131.18 | 105.86 ± 1.85 | 0.807 ± 0.014 | 103.02 ± 1.58 | | 0.785 ± 0.012 | 93.61 ± 1.54 | | 0.714 ± 0.012 |
| Phenylalanine | 165.19 | 38.18 ± 0.52 | 0.231 ± 0.003 | 33.21 ± 0.47 | | 0.201 ± 0.003 | 45.77 ± 0.39 | | 0.277 ± 0.002 |
| Tyrosine | 181.20 | 29.93 ± 0.91 | 0.165 ± 0.005 | 28.10 ± 0.56 | | 0.155 ± 0.003 | 44.95 ± 0.97 | | 0.248 ± 0.005 |
| Methionine | 149.20 | 22.56 ± 0.58 | 0.151 ± 0.004 | 23.41 ± 0.69 | | 0.157 ± 0.005 | 28.45 ± 0.72 | | 0.191 ± 0.005 |
| Cysteine | 240.29 | 20.82 ± 0.46 | 0.087 ± 0.002 | 20.86 ± 0.48 | | 0.087 ± 0.002 | 6.19 ± 0.26 | | 0.026 ± 0.001 |
| Tryptophan | 204.23 | 12.58 ± 0.39 | 0.062 ± 0.002 | 12.77 ± 0.39 | | 0.063 ± 0.002 | 10.72 ± 0.43 | | 0.052 ± 0.002 |
| Threonine | 119.12 | 62.47 ± 1.46 | 0.524 ± 0.012 | 65.56 ± 1.74 | | 0.550 ± 0.015 | 41.24 ± 1.09 | | 0.346 ± 0.009 |
| Lysine | 146.19 | 88.07 ± 1.31 | 0.602 ± 0.009 | 86.42 ± 1.25 | | 0.591 ± 0.009 | 77.53 ± 1.11 | | 0.530 ± 0.008 |
| Aspartic acid | 133.10 | 94.58 ± 1.65 | 0.711 ± 0.012 | 96.64 ± 1.63 | | 0.726 ± 0.012 | 69.69 ± 0.93 | | 0.524 ± 0.007 |
| Serine | 105.10 | 58.13 ± 1.22 | 0.553 ± 0.012 | 50.23 ± 1.05 | | 0.478 ± 0.010 | 51.55 ± 1.28 | | 0.490 ± 0.012 |
| Glutamic acid | 147.10 | 170.93 ± 2.47 | 1.162 ± 0.017 | 185.61 ± 3.02 | | 1.262 ± 0.021 | 217.73 ± 3.45 | | 1.480 ± 0.023 |
| Glycine | 105.10 | 23.43 ± 0.49 | 0.223 ± 0.005 | 18.31 ± 0.55 | | 0.174 ± 0.005 | 17.32 ± 0.34 | | 0.165 ± 0.003 |
| Alanine | 89.10 | 47.72 ± 0.77 | 0.536 ± 0.009 | 48.11 ± 0.57 | | 0.540 ± 0.006 | 31.34 ± 0.38 | | 0.352 ± 0.004 |
| Histidine | 155.20 | 23.43 ± 0.47 | 0.151 ± 0.003 | 17.88 ± 0.49 | | 0.115 ± 0.003 | 25.98 ± 0.51 | | 0.167 ± 0.003 |
| Arginine | 174.20 | 39.48 ± 0.65 | 0.227 ± 0.004 | 25.54 ± 0.67 | | 0.147 ± 0.004 | 32.99 ± 0.53 | | 0.189 ± 0.003 |
| Proline | 115.10 | 55.97 ± 0.63 | 0.486 ± 0.005 | 64.71 ± 1.02 | | 0.562 ± 0.009 | 94.85 ± 1.52 | | 0.824 ± 0.013 |
| *h*_tot_ |  |  | 7.535 ± 0.006 |  | | 7.558 ± 0.062 |  | | 7.470 ± 0.023 |

MFGMP, milk fat globule membrane protein; WPC, whey protein concentrate; MCC, micellar casein concentrate; *h*_tot_ ,the total number of peptide bonds per gram of protein.
